# Suture versus Sutureless Retroperitoneal Robot-assisted Partial Nephrectomy using Veriset™ Hemostatic Patch: A Propensity Score Matched Comparative Analysis from a Single Centre

**DOI:** 10.1590/S1677-5538.IBJU.2026.0143

**Published:** 2026-05-30

**Authors:** Atanu Kumar Pal, Jeni Mathew, Rhea Ranjit Varma, Sandeep Prabhakaran, Kishore TA

**Affiliations:** 1 Aster Medcity Vattikuti fellow of robotic Urologic Surgery Department of Urology Kochi Keral India Department of Urology, Vattikuti fellow of robotic Urologic Surgery, Aster Medcity, Kochi, Kerala; 2 Aster Medcity Department of Urology Kochi Kerala India Department of Urology, Aster Medcity, Kochi, Kochi, Kerala

**Keywords:** Hemostatics, Nephrectomy, Robotic Surgical Procedures

## Abstract

**Objective:**

To evaluate the efficacy of Veriset™ hemostatic patch as a substitute for conventional renorrhaphy in retroperitoneal robot-assisted partial nephrectomy (RAPN).

**Materials and Methods:**

A total of 113 patients underwent retroperitoneal RAPN between January 2024 and January 2025. After propensity score matching on age, BMI, and R.E.N.A.L nephrometry score, 32 and 40 patients underwent Veriset™ placement and renorrhaphy, respectively, were included for final analysis. The clinico-demographic data, R.E.N.A.L. nephrometry score, blood loss, hemoglobin-drop, hospital stay, complications, and renal function on follow-up were prospectively recorded.

**Results:**

The mean age, body-mass-index, and R.E.N.A.L. score were 52.6 ± 9.86 years, 27.41 ± 4.06 kg/m^2^, and 5.24 ± 1.17, respectively. As per the low, intermediate, and high-risk categories of nephrometry, the patients were evenly distributed. The console time and warm ischemia time (WIT) were significantly less in the Veriset group (47.26 ± 17.51 vs. 71.67 ± 22.6 minutes, and 12.86 ± 6.14 vs 17.48 ± 5.40 minutes, respectively, p<0.001). Blood loss was significantly higher in the renorrhaphy group (57.74 ± 22.83 vs. 87.34 ± 33.81 mL, p=0.008). The change in GFR was significantly less in the Veriset group on postoperative day-1 (p=0.008), but became comparable after six months. The postoperative pain score at 6, 12, and 24 hours, blood transfusion, hospital stay, and readmission rates were similar in both groups.

**Conclusion:**

As compared to the renorrhaphy group, Veriset group patients had less console time, WIT, blood loss and immediate reduction of GFR. Sutureless retroperitoneal RAPN can produce good perioperative results without increasing complications.

## INTRODUCTION

Management of small renal masses has evolved toward an individualized approach incorporating active surveillance, nephron-sparing surgery, and ablative therapies (1, 2). Robot-assisted partial nephrectomy (RAPN) remains the most widely adopted and preferred surgical option (3). Since its introduction in 2009, the retroperitoneal approach has offered rapid and direct access to the renal hilum, early identification and control of the renal artery—often encountered before the renal vein—without colon mobilization. Avoidance of intraperitoneal dissection reduces the risk of postoperative ileus and facilitates earlier recovery of bowel function. Additionally, in the patients with prior abdominal surgery, it minimizes the challenges associated with intra-abdominal adhesions (4). Retroperitoneal approach (RAPN) provides excellent perioperative, oncological, and functional outcomes (5). The ‘trifecta outcome’ [warm ischemia time (WIT) less than 25 minutes, negative surgical margins, and the absence of complications] can be consistently attained (6). In recent meta-analyses, retroperitoneal RAPN has shown less blood loss, blood transfusion, complication rates, and length of stay (7, 8). It also achieved satisfactory results in the renal tumours with higher preoperative RENAL scores (9).

Preservation of viable renal parenchyma remains the fundamental principle of partial nephrectomy (PN). Several surgical strategies—such as tumor enucleation, enucleo-resection, off-clamp PN, early unclamping, and selective arterial clamping—have been developed. Renorrhaphy has traditionally been an integral component of PN, serving to achieve hemostasis and maintain parenchymal integrity. However, the compressive forces applied during renorrhaphy may result in strangulation of viable renal tissue and inadvertent ligation of intrarenal parenchymal arteries (10). Furthermore, inner renorrhaphy may injure or lacerate interlobar and segmental vessels, leading to intimal disruption and focal ischemia. The delayed vessel wall erosion, slippage of sliding clips or bolsters, and impaired vascular healing may ultimately result in pseudoaneurysm formation (11). To address these technical limitations, various topical hemostatic agents have been increasingly utilized, as they were associated with reductions in warm ischemia time, operative duration, and estimated blood loss, without an increase in postoperative complications (12).

Veriset™ was developed by Covidien (Mansfield, MA, USA), composed of polyethylene glycol (PEG), tri-lysine, buffer salts, and oxidized cellulose. It has dual mode of action on the tumour bed: it can produce a tamponade effect by giving mechanical compression after getting soaked in the blood due to its self-adhesive hydrogel properties, and at the same time, it concentrates platelets and other clotting factors. The patch gets absorbed within four weeks of application (13). This hemostatic path achieved hemostasis irrespective of bleeding severity or the surface area during hepatic resection (14). Veriset™ also demonstrated good hemostasis in cardiovascular procedures (13).

There has been limited literature regarding the utility of Veriset™ as an alternative to conventional renorrhaphy while performing minimally invasive PN. Few studies have demonstrated the efficacy of this hemostatic patch to control bleeding in transperitoneal partial nephrectomy, but so far, the use of Veriset™ in retroperitoneal PN has not been evaluated. In this article, we analysed the feasibility of Veriset™ in retroperitoneal RAPN and compared it with the conventional renorrhaphy.

## MATERIALS AND METHODS

The study included 113 patients who received retroperitoneal RAPN between January 2024 and June 2025. These patients’ data were gathered in a prospective manner. Ethical clearance was obtained from the Internal Review Board (IRB) of the institute ethics committee (IEC) (Ref. AM/EC/356-2023). The patients with a contralateral normal-functioning kidney and who had a single renal tumour on imaging and were planned for retroperitoneal RAPN were included. The exclusion criteria were patients with numerous renal masses, chronic liver disease, on dual antiplatelets, significant breaches of the pelvicalyceal system (PCS), such as breaches of two or more renal calyces or pelvis. The clinico-demographics, tumour size and location, number of renal vessels, blood parameters before and after surgery, the R.E.N.A.L nephrometry score, intraoperative findings, blood loss, transfusion, post operative course, hospital stay, complications and follow up of 6 months duration along with postoperative serum creatinine and estimated GFR (eGFR) were recorded. For nephrometry, the R.E.N.A.L. score was used, and GFR was assessed using the Modification of Diet in Renal Disease (MDRD) formula. The two groups underwent propensity score matching based on age, body mass index (BMI), and R.E.N.A.L nephrometry score.

## SURGICAL TECHNIQUE

The patient was positioned laterally as per the site of the tumour. Superiorly, the costal margin, inferiorly the anterior superior iliac spine (ASIS), and anterior, mid, and posterior axillary lines were marked. The camera port was placed under vision (Figure-1A). A reniform-shaped balloon was inserted to create the retroperitoneal space under vision. All the retroperitoneal RAPNs were performed using the Da Vinci X and Xi robot (Intuitive Surgical, Sunnyvale, CA, USA) (Figures 1B, 1C).

**Figure 1:**
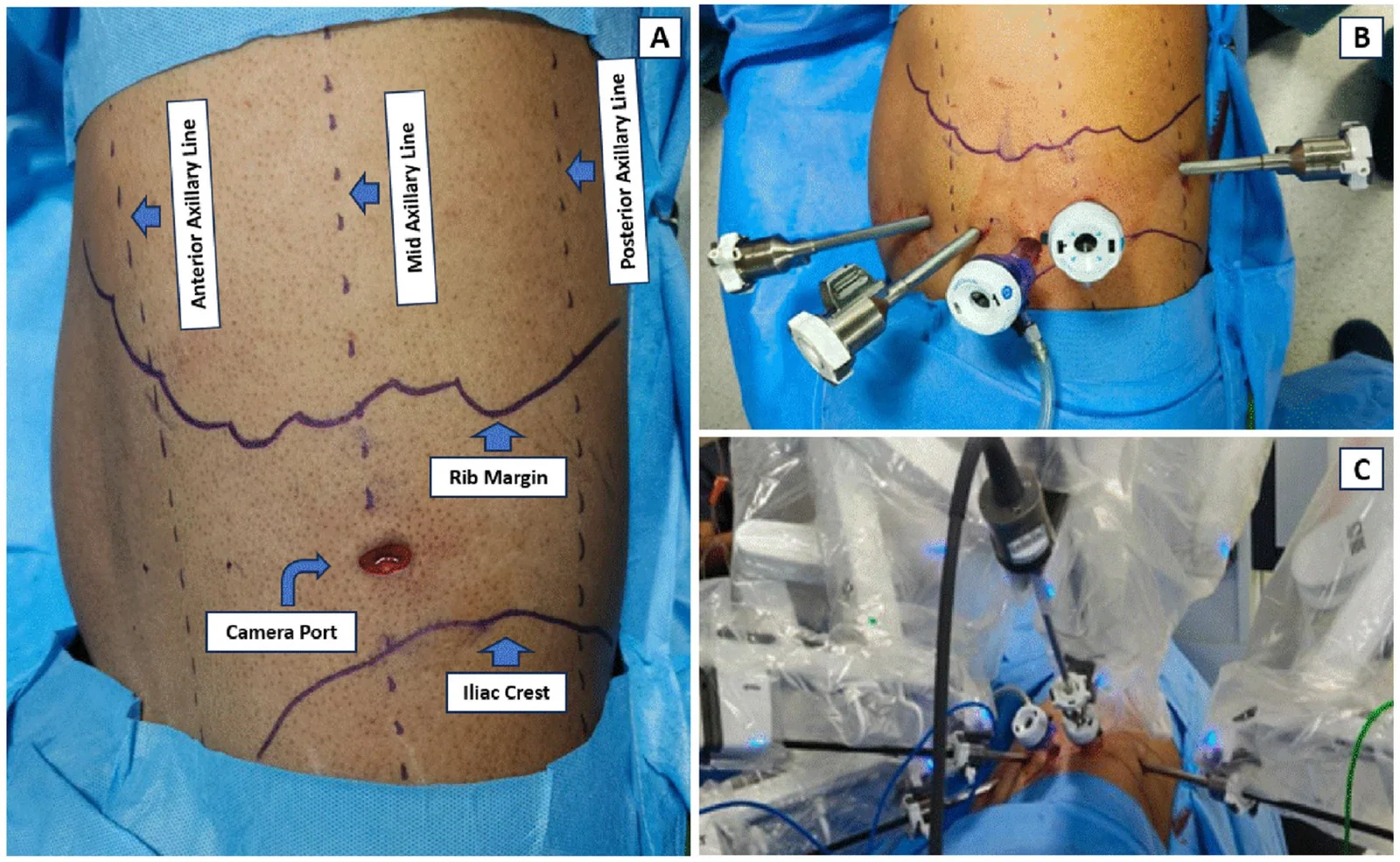
Retroperitoneal port placement.

A) The landmarks and the area of the camera port placement; B) Four robotic and one assistant port placement; C) The final picture after docking the robotic system.

**Veriset™ Group (VG):** The lateroconal fascia was divided to approach the hilum. The renal artery and vein were skeletonised. The tumour was located with intraoperative ultrasonography, dissected, and scored. The artery was clamped with bulldog clamps and the tumour was enucleated (Figure-2A). In some cases, enucleo-resection was performed. Spot coagulation was used on the tumour bed. Stratafix 3-0 was used to seal any pelvicalyceal system breaches (Ethicon, Inc., NJ, USA). After that Veriset™ was placed over the tumour bed and compressed with a moist saline-soaked gauze for 1-2 minutes (Figures 2B, 2C, 2D). Depending on the size of the tumour bed after excision, the patch's dimensions varied from 2 X 4 cm to 5 X 5 cm. In a few cases, two patches were used.

**Figure 2:**
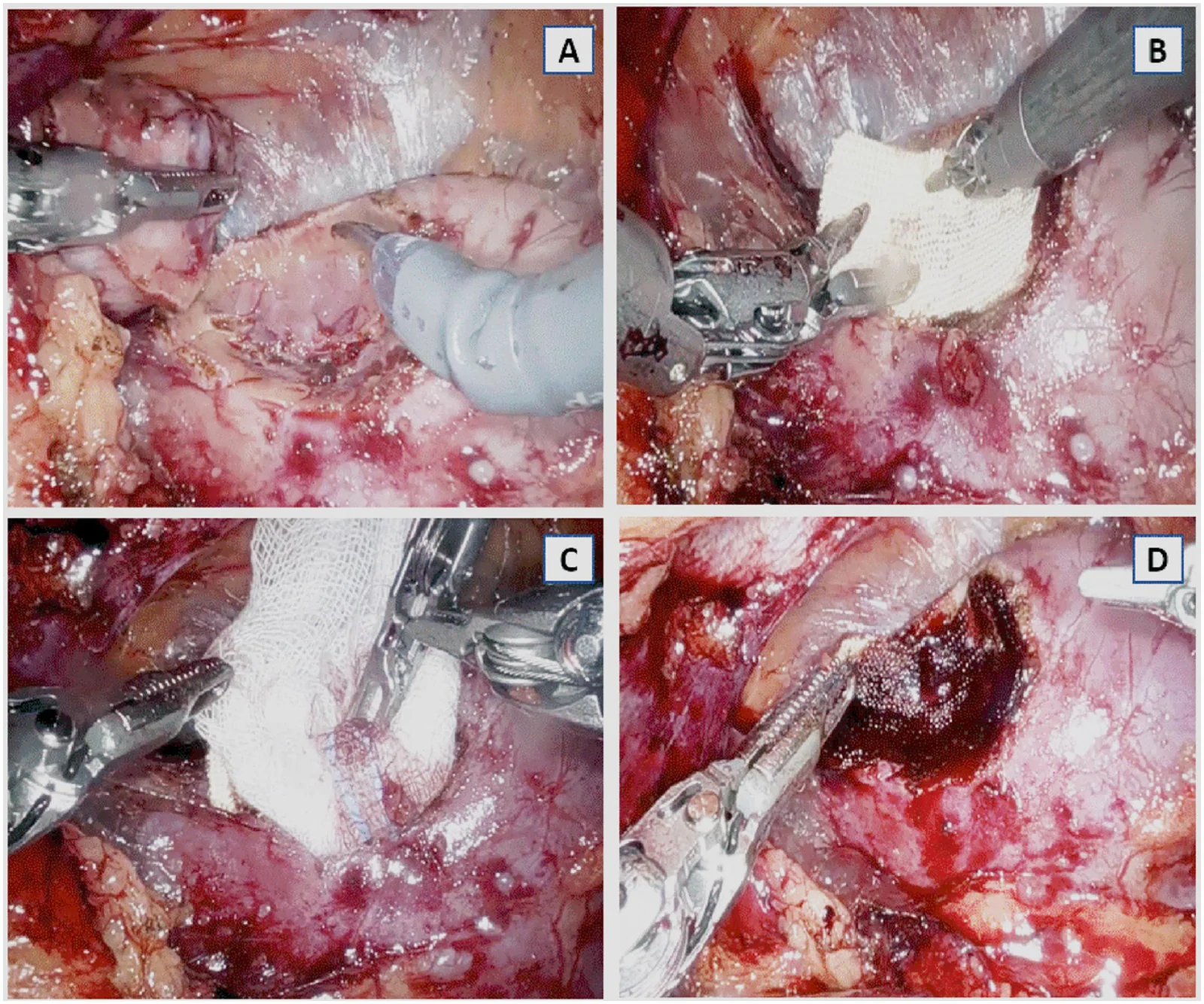
Veriset™ hemostatic patch placement.

A) Tumour bed after enucleation; B) Placement of Veriset™; C) Compression with moist saline gauze; D) Final picture after hemostasis with Veriset™ patch.

**Renorrhaphy Group (RG):** (Figure-3A). Spot coagulation was used after tumour removal (Figure 3A, 3B). The sliding clip technique, renorrhaphy was carried out with a single-layer Vicryl 1-0 (Ethicon, Inc., NJ, USA) (Figures 3C, 3D). No drain was placed in any group of patients.

**Figure 3:**
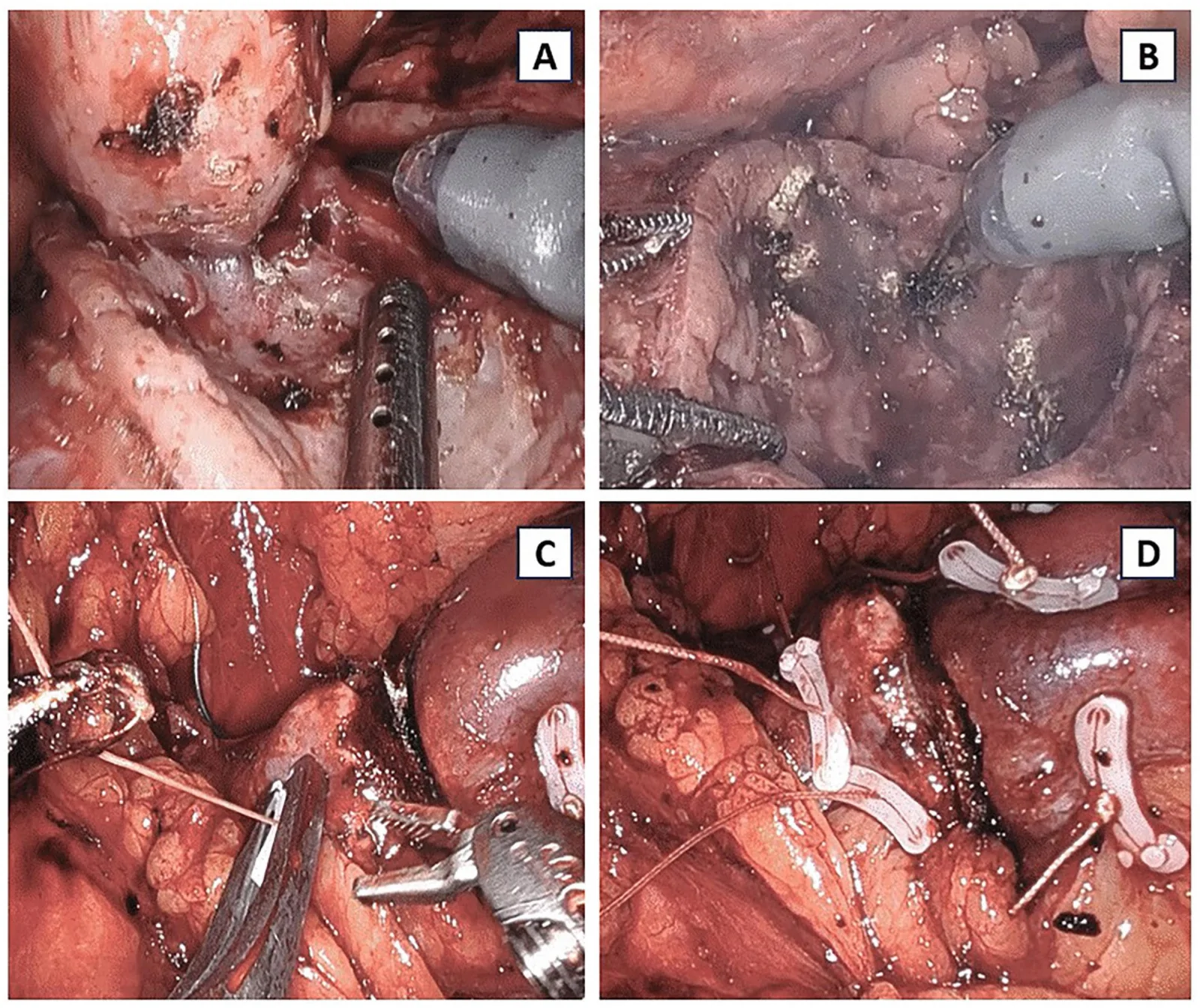
Renorrhaphy.

A) Enucleation of the tumour; B) Spot coagulation of the bleeders on the tumour bed; C) Sliding-clip renorrhaphy; D) Final picture after hemostasis with renorrhaphy.

### Statistical Analysis

R version 4.0.0 (R Foundation for Statistical Computing, Vienna, Austria) was used to conduct the statistical analysis. For continuous variables, descriptive statistics were shown as means with standard deviations; for categorical variables, they were shown as frequencies and percentages. Categorical variables were compared using the Chi-squared test or Fisher's Exact test. Independent sample t-tests were used to compare continuous variables. The statistical significance was considered at a p-value of <0.05.

## RESULTS

A total of 113 patients underwent retroperitoneal RAPN during the study period. Of these patients, 32 patients had Veriset patch placed (VG). Eighty-one patients underwent conventional renorrhaphy (RG). The mean age, BMI, and R.E.N.A.L. nephrometry score were 54.3 ± 12.4 vs. 52.08 ± 15.96 years, 26.95 ±7.24 vs. 27.27 ± 4.26 kg/m^2^, 5.3 ± 1.12 vs. 5.27 ± 1.23, respectively (Table-1).

**Table 1:** Comparison of different perioperative, oncological and renal function parameters between the Veriset™ group (VG) and the renorrhaphy group (RG).

Parameters		Retroperitoneal Robot assisted Partial Nephrectomy		p-value
		Veriset Group (n = 25)	Renorrhaphy Group (n = 40)	
Age (Years)		53.5 ± 10.2	52.08 ± 15.96	0.92
Gender	Male	15 (60%)	28 (75.6%)	0.87
	Female	10 (40%)	9 (24.4%)	
Laterality	Right	11 (44%)	21 (56.7%)	0.59
	Left	14 (56%)	16 (43.3%)	
BMI (kg/m2)		27.84 ±5.12	27.27 ± 4.26	0.9
Tumour Size (cm)		3.32 ± 1.15	3.34 ± 0.9	0.78
Number of Renal Arteries		1.35 ± 0.24	1.43 ± 0.33	0.32
Number of renal Veins		1.46 ± 0.42	1.21 ± 0.18	0.43
Nephrometry Score		5.32 ± 1.29	5.27 ± 1.23	0.75
R.E.N.A.L. Nephrometry Score Category	Low Risk (4-6)	17 (68%)	25 (67.6%)	1.000
	Intermediate Risk (7-9)	6 (24%)	8 (21.6%)	
	High Risk (10-12)	2 (8%)	4 (10.8 %)	
Blood Loss (mL)		59.45 ± 24.64	87.34 ± 33.81	0.008
Blood Transfusion		0 (0.0%)	1 (2.7%)	0.394
Console Time (Minutes)		45.34 ± 16.21	71.67 ± 22.6	<0.001
Warm Ischemia Time (Minutes)		12.73 ± 5.83	17.48 ± 5.40	<0.001
Pain Score- VAS	at 6th hour	1.87 ± 0.43	1.72 ± 0.25	0.56
	at 24th hour	1.63 ± 0.43	1.53 ± 0.42	0.83
Hemoglobin (g/dL)	Preoperative	13.42 ± 4.5	13.81 ± 1.75	0.35
	Postoperative	12.92 ± 1.85	12.66 ± 1.72	0.73
Change in Hemoglobin (g/dL)		-0.95 ± 0.38	-1.15 ± 0.3	0.075
Serum Creatinine (mg/dL)	Postoperative day 1	1.04 ± 0.34	1.16 ± 0.39	0.72
	At 6 Months	1.18 ± 0.27	1.28 ± 0.25	0.67
GFR (mL/min/1.73 m2)	Preoperative	90.43 ± 23.15	87.52 ± 23.28	0.63
	Postoperative day 1	87.44 ± 25.34	83.54 ± 18.89	0.83
	at 6 months	82.78 ± 30.15	80.29 ± 25.63	0.325
Change in eGFR (ml/min/1.73 m2)	Postoperative day 1	-0.07 ± 10.27	-4.68 ± 11.66	0.008
	At 6 Months	-7.01 ± 12.78	-7.68 ± 11.09	0.237
Margins (Positive)		1 (5.0%)	0 (0.0%)	0.567
Length of Stay (Days)		3.25 ± 0.93	3.38 ± 1.28	0.587
Postoperative Hematuria		0 (0.0%)	2 (5.4%)	0.54
Urine Leak		0 (0.0%)	0 (0.0%)	1.000
Pseudoaneurysm		0 (0.0%)	1 (2.7%)	0.394
Angioembolisation		0 (0.0%)	1 (2.7%)	0.394
Hospital Readmission		0 (0.0%)	1 (2.7%)	0.279
Propensity Score		0.25 ± 0.05	0.25 ± 0.05	0.775

BMI = Body mass index; VAS = Visual analogue scale; GFR = Glomerular filtration rate

After propensity score matching, 32 patients in the VG and 40 patients in the RG were taken for the final analysis (Propensity score 0.25 ± 0.05). The tumour size was comparable (3.33 ± 1.2 vs. 3.34 ± 0.9 cm, respectively). As per the R.E.N.A.L. Nephrometry Score Category, low, intermediate, and high-risk tumours were evenly distributed in both groups (65.6 vs. 67.6%, 25 vs. 21.6%, and 9.4 vs. 10.8%, respectively). The warm ischemia time (12.86 ± 6.14 vs. 17.48 ± 5.40 minutes, p<0.001), console Time (47.26 ± 17.51 vs. 71.67 ± 22.6 minutes, p<0.001), and blood Loss (57.74 ± 22.83 vs. 87.34 ± 33.81 mL, p=0.008) were significantly less in the VG. Three patients in the VG required suturing of the large bleeding vessels in tumour bed before patch application. Although hemoglobin drop was less in the VG (0.84 ± 0.21 vs. 1.15 ± 0.3 gm/dL), it was not statistically significant (p=0.075). The immediate drop in GFR on postoperative day-1 was significantly less in the VG (0.07 ± 11.15 vs. 4.68 ± 11.66 mL/min/1.73 m^2^, p=0.008). Although at 6-month follow-up, the change in GFR becomes similar in both groups (-7.21 ± 10.51 vs. −7.68 ± 11.09, mL/min/1.73 m^2^, p=0.237). The postoperative pain score, length of hospital stays, and margin positivity rate were not affected by either group. Overall, two patients in the RG developed hematuria. One of them had readmission and undergone angioembolisation. None of the patients had any urine leak; one patient in the RG had a pseudoaneurysm on follow-up (Table-1).

## DISCUSSION

RAPN is a well-established and feasible minimally invasive approach for renal tumours (15). The transperitoneal approach is still used most commonly as it provides quick access, simple port insertion, a larger workspace with familiar anatomy. However, the retroperitoneal approach has provided non-inferiority in achieving Trifecta outcome along with shorter operating and ischemia times, hospital stays, less intraoperative blood loss, and fewer complications without compromising the GFR (16). Renorrhaphy continues to remain the cornerstone for re-approximation of the renal parenchyma following tumor excision. Techniques like running knotless suturing with Lapra-Ty (absorbable Polyglactin) clips, Suture-clips, Endoclips, or Hem-o-lok clips were invented to minimise the parenchymal compression. Barbed suture, like V-Loc further simplified the renorrhaphy by providing uniform tension distribution. Novel suturing strategies—including V-hilar repair, ring renorrhaphy, and off-clamp renorrhaphy—have been developed (17). Renorrhaphy has been associated with pseudoaneurysm in at least 1-5% patients within the first 90 days after RAPN (18). Gill et al. proposed two mechanisms: firstly, the inadvertent vascular injury during suturing, and secondly, the increased blood flow and accumulation at the injured site after the patient resumes daily activity (19). Renorrhaphy, along with excision of viable renal parenchyma and prolonged WIT causing ischemia-perfusion damage, can decrease up to 20% of the renal function (20).

To overcome these challenges, the use of various hemostatic agents has been suggested. Grossly four types of agents have been explored: mechanical agents like bovine collagen, polysaccharide spheres, cellulose, and gelatin (Bioglue) form a matrix over the tumour bed and activate the extrinsic coagulation pathway. The active substance, like thrombin, works on the intrinsic pathway to convert fibrinogen into fibrin. The flowable agents (gelatin-based, e.g., Surgiflo, FloSeal, Flogel, and collagen-based, like Collastat) swell after being sprayed from a syringe and create a tamponade effect. Lastly, fibrin based like sealants, foams, and glue (Tachosil: fibrin and thrombin) promote fibrin mesh formation at the end of the coagulation cascade (15). Although initial experience with hemostatic agents was not encouraging for blood loss and hemoglobin drop in RAPN (21). However, recently only 4.4% patients were reported to have failure with Hemopatch (Baxter) (22). Tiscione et al. reported a six-minute and 18-minute reduction in median WIT and operating time respectively, without affecting the renal function (23). Improved functional (operative time [110 vs 150 min; p < 0.01] and length of stay [2 vs 3 d; p = 0.02] and trifecta safety (86% vs. 70%, p=0.1) were seen by Farinha et al. during transperitoneal RAPN when they compared the selective-suturing or sutureless technique with Veriset and Tachosil with standard renorrhaphy (24).

Veriset™ (Covidien; Mansfield, MA, USA) is composed of absorbable oxidized cellulose and hydrogel components without any human or animal component. Along with providing mechanical compression by forming a cross-linked PEG-based gel while interacting with blood, it can concentrate platelets and other clotting factors over the tumour bed to accelerate coagulation (13). The oxidised cellulose may provide anti-microbial cover. It requires at least 30 seconds of pressure with a moist saline gauze and gets absorbed after four weeks of application. It is very easy to insert through the assistant port. As described by Brassetti et al., we perform high-precision coagulation of the bleeders on the tumour bed before placing the Veriset™ (20). To lower WIT and postoperative pseudoaneurysm rates, we have changed our practice from two-layer renorrhaphy to single-layer outer cortical renorrhaphy (SLOCR) (25). Our study result still shows significantly less WIT (12.86 ± 6.14 vs. 17.48 ± 5.40 minutes, p<0.001), console Time (47.26 ± 17.51 vs. 71.67 ± 22.6 minutes, p<0.001) in the VG. Erne et al. found similar results, with 35 and six minutes less mean operating time and WIT, respectively, in the patients who underwent laparoscopic partial nephrectomy (26). In our patients of VG, we released the arterial clamp immediately after the patch placement at the start of the compression, while in RG, we only released it after the renorrhaphy, which we feel attributed to the shorter WIT in the VG.

In our study, VG had significantly less blood Loss (57.74 ± 22.83 vs. 87.34 ± 33.81 mL, p=0.008). Although the hemoglobin drop was less in VG, it was not significant. Maurice et al. recorded 2.4% blood transfusions. After covariate adjustment, they found no alteration in postoperative bleeding outcome by the hemostatic agents in nonanemic patients (27). However, in our propensity-matched analysis between VG and RG in transperitoneal RAPN, we found less blood loss in VG (28). Guo et al. found a similar result of almost 80 mL less mean estimated blood loss (12). As the retroperitoneum is a less distensible compartment compared to the peritoneal cavity, the pneumoperitoneum can create more compression on the tumour bed. This effect helps in more precise resection or enucleation of the tumour, and Veriset patch, along with pneumoperitoneum, secures the hemostasis in a more efficient manner. Two patients in RG had hematuria. One of them had readmission and was diagnosed to have pseudoaneurysm formation and underwent angioembolisation. None of the patients had such an outcome in the VG. In our earlier study evaluating the use of Veriset™ in transperitoneal robot-assisted partial nephrectomy, three failures were observed, necessitating additional renorrhaphy. These failures occurred due to bleeding following clamp release, which was attributed to overlooked arteries within the tumor base. In contrast, no failures were encountered in the retroperitoneal group. This difference may be explained by improved visualization of intratumoral arteries during tumor resection in the retroperitoneal approach, facilitated by effective tamponade of small renal veins, resulting in a clearer surgical field.

Renorrhaphy produces parenchymal ischemia and damage, and thereby produces a reduction in eGFR. In our study, the immediate rise in serum creatinine and eGFR drop was more pronounced in the RG, but this effect faded with time, and the renal function became comparable between both groups at 6 months. This could be explained by the fact that the WIT in both our study groups was less than 20 minutes, which led to such a functional outcome. In a recent analysis, duration of ischemia did not affect the renal function in RAPN if the WIT was below 30 minutes (29).

The Veriset™ patch was used across all R.E.N.A.L. nephrometry grades. The mean tumour size in both groups was similar (3.33 ± 1.2 vs. 3.34 ± 0.9 cm, respectively). Not any patient in these two groups suffered from urine leak, which signifies proper watertight closure of the pelvicalyceal system. Neither approach resulted in significant margin positivity. Complication rates were also comparable. Erne et al. recorded a similar outcome in their patients after laparoscopic PN (26). Based on these findings, Veriset is non-inferior in achieving the ‘Trifecta outcome’ while compared to renorrhaphy.

Hemostatic agents are associated with additional cost. Durant et al. analysed that the cost difference with renorrhaphy was greatest during 2010 (30). However, as Veriset™ hemostatic patch eliminates the need for consumables needed for renorrhaphy, i.e., sutures and hemo-lock clips, our rough estimation suggests that the cost of clips and sutures is largely comparable to a single Veriset patch, with costs increasing only when multiple patches are required. The cost factor warrants re-evaluation, and a detailed comparative analysis is required to understand the economic impact.

The indications for conventional renorrhaphy and hemostatic patch placement should be clearly defined. Veriset™ may be particularly advantageous where minimizing WIT and preserving viable renal parenchyma are of paramount importance, such as in patients with multiple renal tumors, syndromic conditions including von Hippel–Lindau disease (VHL), a solitary kidney, prior contralateral nephrectomy, or pre-existing chronic kidney disease. Conversely, as Veriset™ relies on an intact coagulation pathway to achieve optimal efficacy, its use should be avoided in patients with chronic liver disease, bleeding diathesis, or clotting factor deficiencies. In addition, cases with extensive pelvicalyceal system breach or a grossly irregular tumor bed following resection may not be suitable for this approach.

There are a few limitations in this study. Firstly, the sample size is small, and from a single centre. With such a small sample size, the study lacks the necessary statistical power (Type II error) to generalise the conclusion of no difference between the two techniques. Secondly, this non-randomised study can give rise to selection bias. Although propensity scores were matched, confounding factors cannot be ruled out. Thirdly, as we initially adopted Veriset™ in retroperitoneal RAPN, its use was largely limited to tumors with low nephrometry scores, resulting in a relatively small proportion of high-complexity cases in our cohort. Therefore, drawing a definitive conclusion regarding its safety or efficacy in the high nephrometry score subgroup is not feasible. The follow-up was short without any formal cost analysis. We avoided Veriset™ in major PCS breaches, which may have introduced a selection bias. Despite these, this is the first study that did a head-to-head perioperative, oncological, and functional outcome comparison of Veriset™ hemostatic patch with renorrhaphy in retroperitoneal RAPN.

## CONCLUSIONS

This study indicates that Veriset™ hemostatic patch during retroperitoneal RAPN can be considered as a safe and feasible alternative to conventional cortical renorrhaphy to achieve hemostasis and the trifecta outcome. It can bring down warm ischemia time, console time, and blood loss without increasing the complications and reducing the renal function. However, as the study population predominantly comprised low nephrometry score tumors, the role of this approach in moderate- and high-complexity renal masses remains to be established and warrants validation in larger studies.

## Data Availability

Trial data will be made available on reasonable request to the corresponding author.
